# Robotic Sensing and Systems for Smart Cities

**DOI:** 10.3390/s21092963

**Published:** 2021-04-23

**Authors:** Hyun Myung, Yang Wang

**Affiliations:** 1 Korea Advanced Institute of Science and Technology, School of Electrical Engineering, 291 Daehak-ro, Daejeon 34141, Korea; 2 Georgia Institute of Technology, School of Civil and Environmental Engineering, 790 Atlantic Drive, Atlanta, GA 30332-0355, USA; yang.wang@ce.gatech.edu; 3 Georgia Institute of Technology, School of Electrical and Computer Engineering (Adjunct), 790 Atlantic Drive, Atlanta, GA 30332-0355, USA

## 1. Introduction

For several decades, various sensors and sensing systems have been developed for smart cities and civil infrastructure systems. This Special Issue focuses on state-of-the-art robotics and automation technologies, including construction automation, robotics, instrumentation, monitoring, inspection, control, and rehabilitation for smart cities. It also covers construction informatics supporting the sensing, analysis, and design initiatives needed to construct and operate smart and sustainable built environment.

Examples include robotic systems adopted for smart cities and equipped with various sensing technologies, such as vision-based sensors, laser sensors, wireless sensors, multi-sensor fusion, etc. Service robots and robotized devices for increasing the usability and safety of built environment are also appropriate examples.

A Special Issue related to these areas is being published in an effort to share the current advances of various robotics and automation technologies for smart cities.

## 2. Summary of the Special Issue

The work developed in [1] presents a UAV (unmanned aerial vehicle) system for autonomously performing electrical and mechanical (E+M) device inspection. The proposed system relies on learning-based detection for perception, multi-sensor fusion for localization, and path planning for fully autonomous inspection. The perception method utilizes semantic and spatial information generated by a 2D object detector YOLO-v4. Based on visual–inertial odometry framework, the information is then fused with stereo camera-based depth measurements for object state (pose) estimation. The proposed system is validated by flight experiments using a quadrotor platform. They inspected three objects, including a traffic light, a bulb, and CCTV. The result shows that the proposed UAV system enables the autonomous inspection mission and ensures a stable and collision-free flight, which will be useful for the inspection of smart city infrastructures.

The work in [2] deals with autonomous robot inspection tasks of water distribution pipes. They presented a novel method for improving the estimation of a robot’s trajectory using the measured acoustic field in the pose-graph optimization framework. Experimental results show that the use of acoustic information in pose-graph optimization reduces errors by 39% compared to those of landmark features only. High location accuracy promotes effective target investment to maximize the use of aging pipe infrastructure in smart cities.

The study in [3] proposed a method for evaluating road safety by analyzing point cloud data collected by a mobile scanner. They proposed a novel geometric visual model to measure drivers’ visual perception and analyze the corresponding information using the line-of-sight method. This study was motivated by the fact that a driver generally obtains visual information while driving, and this information is directly related to traffic safety in smart cities. To investigate drivers’ visual perceptions of roads, they developed an analytic model of three types of visual perception depending on the vehicle’s speed. By analyzing point cloud data with the proposed method, they verified that trees and obscuring objects along a road affected drivers’ visibility. By using this proposed method, this study creates a risk-level map according to the driver’s visual perception degree in a specific city in South Korea. With the point cloud data of the city, it was possible to analyze actual urban forms such as roadside trees, building shapes, and overpasses that were normally excluded from spatial analyses that use a reconstructed virtual space.

The study in [4] presents a novel framework that integrates the deep learning technique and state-machine control to develop a reliable and repeatable human-following robot. People are detected and tracked using SSD (single shot detector). The target person is identified by extracting the color feature using an HS (hue, saturation) histogram from a video sequence. The robot follows the target safely using LiDAR-based SLAM (simultaneous localization and mapping). They designed an efficient and repeatable robotic state-machine control so that the robot stays in an active state of the human following without freezing. They adopted a robust vision algorithm from occlusions and illumination changes integrating a color feature with the deep learning algorithm. They developed a robust human following and verified it in realistic indoor situations.

The work in [5] proposes a novel mobile robot self-localization based on an onboard 2D push-broom (or tilted-down) LiDAR using a reference 2D map previously obtained with a 2D horizontal LiDAR. The motivation to tilt-down a 2D LiDAR is the direct detection of holes or small objects placed on the ground that remain undetected for a fixed horizontal 2D LiDAR. The experiments demonstrated that self-localization with a 2D push-broom LiDAR is possible by detecting and deleting the ground and ceiling points from the scan data and projecting the remaining scan points in the horizontal plane of the 2D reference map before applying a 2D self-location algorithm. The use of low-cost 2D LiDARs instead of expensive 3D LiDARs for autonomous navigation purposes will promote various applications of mobile robots in smart cities.

The study in [6] presents an integrated human–robot interaction system architecture. One of the main differences between the proposed architecture and other ones is the information acquisition method regarding the robot’s interlocutor. In order to obtain as much information as possible before the actual interaction took place, a custom internet-of-things-based sensor subsystems connected to smart infrastructure was designed and implemented. The development supports the interlocutor identification and acquisition of initial interaction parameters. The artificial intelligence interaction framework of the developed robotic system is being extended with the use of custom external subsystems for additional knowledge acquisition: device-based human identification, visual identification and audio-based interlocutor localization subsystems. The evaluation of these subsystems demonstrates the benefits of integrating them into the robotic interaction system.

The work in [7] proposes an inexpensive, lighter, safer, and smaller radar system than military-grade radar systems, while keeping reasonable capability for use in monitoring public places. They presented a compact and cost-effective mobile 2.4 GHz radar system for moving object detection. The paper details the iterative process on the system design and improvements with experiments to realize the system used for surveillance. The experiments show the practical use of the system and configuration for a better understanding of using the system. The developed system is expected to be useful for surveillance systems in public spaces that need to protect personal privacy.

A technical note in [8] presents a new virtual reality toolkit named LoopAR for Unity applications investigating human–machine interaction in highly automated driving scenarios. The presented setup is a mobile, cost-efficient, and highly adaptable alternative to chassis simulators that closely monitor the participants. There is not only a drastic reduction in costs, but also an improvement to the adaptability of the software, as well as the used hardware. LoopAR allows interested researchers to conduct various virtual reality experiments without creating the needed environment or functionalities themselves. The authors provided an area of almost 25 km^2^. The toolkit also includes all the necessary assets and basic prefabs to quickly and precisely create a wide variety of virtual environments. The LoopAR toolkit is expected to be useful for various simulations in smart cities.

## 3. Conclusions

This Special Issue consists of eight papers with various application domains surrounding smart cities. These include UAVs, mobile robots, inspection robots, autonomous vehicles, human–robot interaction systems, and sensor systems. Future smart cities will benefit from all these affordable technologies. The intensive collaborations among companies, universities, and research institutes are essential for implementing sustainable smart cities due to the necessity for multidisciplinary research and development.

Finally, we would like to thank all authors who have submitted their manuscripts to this Special Issue of *Sensors* and the reviewers for their efforts during the review process.

## Data Availability

Not applicable.
